# Supplementary material for: Dietary Leucine Improves Fish Intestinal Barrier Function by Increasing Humoral Immunity, Antioxidant Capacity, and Tight Junction
Source: Int J Mol Sci. 2023 Mar 1;24(5):4716. doi: 10.3390/ijms24054716 (PMC10003359; doi:10.3390/ijms24054716)
Supplement: Supplementary file 1 [file ijms-24-04716-s001.zip › ijms-2044066-supplementary.pdf]

Table S1. Composition and nutrients content of basal diet.

Ingredients	g/kg dry diet	Nutrients content[a]	g/kg dry diet
Fish meal	170.0	Crude protein	384.7
Casein	10.0	Crude fat	71.0
Gelatin	10.0	Available phosphorus	14.8
Amino acid premix[b]	170.0	ω-3	15.2
Leucine-glycine premix[c]	120.0	ω-6	13.9
α- starch	210.0		
Corn starch	168.3		
Fish oil	30.0		
Soybean oil	26.0		
Vitamin premix[d]	10.0		
Mineral element premix[e]	20.0		
$Ca(H_2PO_4)_2$	40.0		
Choline chloride (50%)	10.0		
Ethoxyquin (30%)	0.5		
Cellulose	5.0		
XanthophyII	0.2		

[a] The values of crude protein, crude fat, and crude ash were measured. Available phosphorus, n-3 and n-6 contents were calculated according to NRC (2011). [b] Amino acid mix (g/kg): lysine, 125.204; histidine, 27.508; isoleucine, 39.425; arginine, 116.491; methionine, 60.071; cystine, 11.432; phenylalanine, 77.364; threonine, 83.764; tryptophan, 11.723; valine, 11.302; all ingredients were diluted with corn starch to 1 kg. [c] Leucine–glycine premix composition from diet 1 to 7 was as follows (g/kg): L-leucine 0.0, 41.7, 83.3, 125.0, 166.7, 208.3, 250.0; glycine 152.8, 127.3, 101.9, 76.3, 50.9, 25.5, 0.0; corn starch 847.2, 831.0, 814.8, 798.7, 782.0, 766.2, 750.0, respectively. [d] Vitamin premix (g/kg): retinyl acetate (500,000 IU/g), 8.063; cholecalciferol (500,000 IU/g), 0.100; DL-α-tocopherol acetate (500 g/kg), 53.600; menadione (230 g/kg), 0.217; cyanocobalamin (10 g/kg), 0.100; D-biotin (20 g/kg), 5.000; folic acid (960 g/kg), 0.521; thiamin nitrate (900 g/kg),

0.111; ascorbyl acetate (930 g/kg), 86.022; niacin (990 g/kg), 3.143; mesoinositol (990 g/kg), 52.323; calcium-D-pantothenate (900 g/kg), 1.667; riboflavine (800 g/kg), 1.125; pyridoxine hydrochloride (810 g/kg), 0.370; all ingredients were diluted with corn starch to 1 kg. [e] Mineral premix (g/kg): $FeSO_4 \cdot H_2O$ (300 g/kg Fe), 13.333; $CuSO_4 \cdot 5H_2O$ (250 g/kg Cu), 1.300; $ZnSO_4 \cdot H_2O$ (345 g/kg Zn), 13.043; $MnSO_4 \cdot H_2O$ (318 g/kg Mn), 4.717; KI (38 g/kg I), 1.447; $NaSeO_3$ (10 g/kg Se), 1.000; $MgSO_4 \cdot H_2O$ (150 g/kg Mg), 133.333. All ingredients were diluted with $CaCO_3$ to 1 kg.

Table S2. Amino acid composition of the experimental diets (g/kg dry diet) and fish muscle (relative to 384.7 g/kg protein)[1].

Amino acid	Dietary Leu level (g/kg diet)							384.7 g/kg muscle protein
	10.0	15.0	20.0	25.0	30.0	35.0	40.0	
Essential amino acid								
Threonine	1.93	1.89	1.95	1.94	1.90	1.87	1.93	1.94
Valine	1.34	1.38	1.41	1.36	1.39	1.42	1.43	1.40
Methionine	1.26	1.25	1.19	1.23	1.23	1.25	1.22	1.21
Isoleucine	1.17	1.14	1.12	1.16	1.20	1.18	1.17	1.18
Leucine	1.11	1.48	2.02	2.52	3.04	3.57	4.02	2.53
Phenylalanine	1.81	1.79	1.80	1.82	1.75	1.83	1.76	1.77
Histidine	0.84	0.76	0.80	0.82	0.79	0.81	0.85	0.83
Lysine	2.61	2.59	2.62	2.57	2.65	2.58	2.55	2.62
Arginine	2.57	2.55	2.47	2.60	2.58	2.61	2.49	2.53
Tryptophan	0.49	0.56	0.54	0.51	0.55	0.50	0.53	0.52
Nonessential amino acid								
Aspartic acid	4.41	4.50	4.62	4.55	4.48	4.68	4.60	4.56
Serine	1.91	1.93	1.87	1.95	2.01	1.94	2.05	1.97
Glutamic acid	6.82	6.77	6.68	6.71	6.75	6.69	6.66	6.62
Glycine	2.22	2.28	2.26	2.30	2.32	2.29	2.25	2.21
Alanine	2.73	2.85	2.80	2.78	2.71	2.90	2.87	2.82
Cystine	0.21	0.20	0.19	0.25	0.18	0.21	0.17	0.23
Tyrosine	1.51	1.51	1.47	1.45	1.55	1.56	1.49	1.50
Proline	1.44	1.48	1.51	1.57	1.48	1.50	1.53	1.46

[1] Values are means from duplicate samples of experimental diets and fish muscle.

Table S3. IBW, FBW,PWG, SGR, FI, FE, and PE of hybrid catfish fed diets containing graded levels of Leu (g/kg) for 8 weeks.[1]

Items[2]	Dietary Leu levels, g/kg							$Pr > F$[2]		
	10.0	15.0	20.0	25.0	30.0	35.0	40.0	ANOVA	Linear	Quadratic
IBW	23.21 ± 0.13	22.98 ± 0.14	22.90 ± 0.20	23.19 ± 0.27	23.32 ± 0.24	23.30 ± 0.25	23.34 ± 0.19	0.67	0.21	0.49
Survival	96.67 ± 1.93	96.67 ± 0.00	95.56 ± 2.94	98.89 ± 1.11	95.56 ± 1.11	97.78 ± 2.22	94.44 ± 2.93	0.80	0.23	0.16
FBW	43.73 ± 0.70[b]	44.73 ± 0.13[b]	48.22 ± 1.32[b]	54.55 ± 0.87[a]	49.48 ± 1.58[ab]	47.82 ± 2.22[b]	45.53 ± 1.17[b]	0.00	0.08	<.0001
PWG	88.35 ± 1.81[c]	94.70 ± 1.95[bc]	111.20 ± 5.51[bc]	137.89 ± 3.56[a]	117.91 ± 11.96[ab]	112.42 ± 8.26[bc]	94.94 ± 2.99[bc]	0.00	0.31	0.17
SGR	1.13 ± 0.02[b]	1.19 ± 0.02[b]	1.33 ± 0.05[ab]	1.55 ± 0.03[a]	1.34 ± 0.07[ab]	1.27 ± 0.08[b]	1.19 ± 0.03[b]	0.00	0.19	<.0001
FI	37.57 ± 1.19[ab]	38.60 ± 0.54[ab]	42.04 ± 1.84[a]	43.30 ± 3.74[a]	38.00 ± 0.57[ab]	34.25 ± 1.02[bc]	31.66 ± 1.74[c]	0.00	0.00	0.00
FE	53.05 ± 1.42[b]	56.87 ± 2.79[b]	59.08 ± 1.15[b]	74.87 ± 2.67[a]	71.85 ± 4.15[a]	69.01 ± 4.27[a]	67.34 ± 1.48[a]	0.00	<.0001	<.0001
PER	2.06 ± 0.03[b]	2.09 ± 0.07[b]	2.41 ± 0.03[ab]	3.11 ± 0.37[a]	2.53 ± 0.21[ab]	2.52 ± 0.08[ab]	2.58 ± 0.01[ab]	0.02	0.09	0.54

[1]Values are means ± SEM (n = 3, 30 fish in each replicate). Mean values with different superscripts in the same row are significantly different (*P* < 0.05). Initial body weight (IBW, g/fish), survival, final body weight (FBW, g/fish), percent weight gain (PWG, %), specific growth rate (SGR, %/d), feed intake (FI, g/fish), feed efficiency (FE, %), and protein efficiency ratio (PER).

[2]PWG =weight gain (g) / initial weight (g) × 100; SGR = (ln FBW-ln IBW)/d× 100; FE = weight gain (g) / feed intake (g) × 100; PER = weight gain (g) / protein intake (g)

Table S4. Primer sequences and optimal annealing temperatures (OAT, ˚C) of genes selected for analysis by real-time PCR.

Name	Sequences	OAT	Accession number
Intl1-QF	CCCTCTATTCCCGTAGTGTATGATG	63.3	MK562428
Intl1-QR	CCAGGCTTGCTTCTCGCTCT		
Intl2-QF	CTGGGCAGACACCGCAAT	61.4	MK562429
Intl2-QR	AGGACCGCTATCCGCTTTA		
c-LZM-QF	TGCTAATGGGTGTAAAATCTCCT	59.0	KP210247.1
c-LZM-QR	TAATGCCCTGCTGTCTCACTATG		
g-LZM-QF	TGCTAATGGGTGTAAAATCTCCTG	63.3	KP210248.1
g-LZM-QR	CGGTAATGCCCTGCTGTCTC		
β-defensin-QF	CCTACAGAACTACCCTTTGGACC	61.4	KY091312.1
β-defensin-QR	CACAGCAAACAAACCCTTTAGAG		
CuZnSOD-QF	TCACTTCAACCCCCACAACA	63.3	KX455916.1
CuZnSOD-QR	CGGCAGTCACATTACCCAGAT		
CAT-QF	ACACCGATGAGGGAAACTGG	58.0	KX455919
CAT-QR	GTGGATGAAGGACGGGAACA		
GPX1a-QF	GTGAATGGGAAAGACGCTC	61.7	MG773203
GPX1a-QR	GCACACAGGACTCCAGATGA		
GST-QF	CGGATGGGAAATGGAACG	58.0	XM_027154055.1
GST-QR	GGATAATGCTCCTGACTCAACC		
GCLC-QF	GACAAACGGAGGAAGGAGG	58.2	KX455918
GCLC-QR	TCATCAGGAAAGAAGAGGGACT		
Keap1-QF	GCATCCTCTTCACCTGTCT	61.7	MG773201
Keap1-QR	CGTGTAGGCGAACTCTATC		
Nrf2-QF	CGGAACAAGATGGAGAAGCC	64.0	KX455917
Nrf2-QR	ACAGGGAGGAATGGAGGGA		
Beclin1-QF	CTCAACTGGACCGCCTGAAGAAA	59.0	XM_027177667.1
Beclin1QR	CACTCCACAGGAACGCTGGGTAAT		
ULK1b-QF	GGAGGGTGTTGTGATGTTTGAGGC	59.0	XM_027176183.1

ULK1b-QR	CTTGCTGGTGTAGGCAGGTTGTG		
ATG5-QF	CAGAACCGTTTTATCTTCTCCTACCG	59.0	XM_027142793.1
ATG5-QR	CGTCTACATCTTCAGCTTTCACGACTT		
ATG7-QF	CTCCTATTCAAACCCAGTGCGTCAG	65.0	XM_027140729.1
ATG7-QR	ATCAGTGCCTCCAACTGCTCCAC		
ATG9a-QF	CTGCCCATCCGTTTCCGTTTACC	59.0	XM_027160018.1
ATG9a-QR	TTCGGACTTCAGACTCCATTCGTTT		
ATG4b-QF	GCTGCGATGTGGACAGATGATTC	65.0	XM_027147236.1
ATG4b-QR	TCTTGTCTATGAAGGCATTGAGGATGT		
LC3b-QF	CCTGACCACGTCAACATGAGCGAACT	65.0	XM_027140826.1
LC3b-QR	GGAAATGGCGGCAGACACGGAGA		
P62-QF	TTTCTCAAACCTCCAAAATGTCGC	63.3	KY062772
P62-QR	GGGAAGTCACGCTTGTGCTCCTT		
occludin-QF	AGTGTGACTGGAATGGGCG	61.7	MG773197
occludin-QR	CCTCCTGAGGATCGACGTAG		
ZO-1-QF	GCAGTCGCTCTCGTTCCCCA	63.0	MG773194
ZO-1-QR	CCCAGTCTGTTCCAGTGCTTTATGA		
Claudin-2-QF	GTCCAGCGTTGGGATGCA	58.6	MG773206
Claudin-2-QR	GGAAACAGGAATGAGCGACAG		
18S-QF	CCTGAGAAACGGCTACCACATCC	57.1	KP938527
18S-QR	AGCAACTTTAATATACGCTATTGGAG		
β-actin-QF	CCTAAAGCCAACAGGGAAAA	59.0	EU161066
β-actin-QR	ATGGGGCAGAGCATAACC		
